# Increasing late stage colorectal cancer and rectal cancer mortality demonstrates the need for screening: a population based study in Ireland, 1994-2010

**DOI:** 10.1186/1471-230X-14-92

**Published:** 2014-05-13

**Authors:** Nicholas Clarke, Joseph McDevitt, Patricia M Kearney, Linda Sharp

**Affiliations:** 1 National Cancer Registry, Building 6800, Cork Airport Business Park, Kinsale Road, Cork, Ireland; 2 Department of Epidemiology and Public Health, Fourth Floor, Western Gateway Building, University College Cork, Cork, Ireland

**Keywords:** Colorectal, Cancer, Ireland, Incidence, Survival, Mortality, Mass screening, Colon, Rectal

## Abstract

**Background:**

This paper describes trends in colorectal cancer incidence, survival and mortality from 1994 to 2010 in Ireland prior to the introduction of population-based screening.

**Methods:**

We examined incidence (National Cancer Registry Ireland (NCRI) and mortality (Central Statistics Office) from 1994 to 2010. Age standardised rates (ASR) for incidence and mortality have been calculated, weighted by the European standard population. Annual percentage change was calculated in addition to testing for linear trends in treatment and case fraction of early and late stage disease. Relative survival was calculated considering deaths from all causes.

**Results:**

The colorectal cancer ASR was 63.7 per 100,000 in males and 38.7 per 100,000 in females in 2010. There was little change in the ASR over time in either sex, or when colon and rectal cancers were considered separately; however the number of incident cancers increased significantly during 1994-2010 (1752 to 2298). The case fractions of late stage (III/IV) colon and rectal cancers rose significantly over time. One and 5 year relative survival improved for both sexes between the periods 1994-2008. Colorectal cancer mortality ASRs decreased annually from 1994-2009 by 1.8% (95% CI -2.2, -1.4). Rectal cancer mortality ASRs rose annually by 2.4% (95% CI 1.1, 3.6) and 2.8% (95% CI 1.2, 4.4) in males and females respectively.

**Conclusions:**

Increases in late-stage disease and rectal cancer mortality demonstrate an urgent need for colorectal cancer screening. However, the narrow age range at which screening is initially being rolled-out in Ireland means that the full potential for reductions in late-stage cancers and incidence and mortality are unlikely to be achieved. While it is possible that the observed increase in rectal cancer mortality may be partly an artefact of cause of death misclassification, it could also be explained by variations in treatment and adherence to best practice guidelines; further investigation is warranted.

## Background

Over 1.23 million colorectal cancers are diagnosed worldwide annually [1] with 609 000 deaths [1]. Colorectal cancer is highly preventable if diagnosed early and treated. Screening has been available for many years through several modalities, including colonoscopy, sigmoidoscopy, and faecal-based tests [2-4]. Faecal-based tests, notably faecal occult blood testing (FOBT), are generally the route through which colorectal cancer screening programmes are being delivered internationally [5,6]. More recently faecal immunochemical testing (FIT) has been recommended for screening due to its improved sensitivity and specificity in detecting human haemoglobin and the fact that there is no need for test recipients to undergo dietary restrictions (which may be required for guaiac-based tests). Studies which have used FIT suggest improved uptake compared to other screening tests such as FOBT, possibly due to the absence of dietary restrictions, the need for fewer samples, absence of the need for storage if a one sample test, and ease of use [7]. However the authors state that these results are inconclusive and require further investigation from the patient’s perspective [7]. Recent European and US guidelines recommend FIT as the initial screening test in population-based screening programmes [8,9].

Screening aims to detect colorectal disease either at a precancerous stage (when removal of polyps may prevent cancers developing) or when cancers are at an early stage (when treatment is more effective and patients may also benefit from improved quality-of-life). Screening therefore has the potential to reduce mortality, provided the service is of high quality and coverage is high [8].

Although many European countries have established screening programmes, until 2013, no programme was in place in Ireland. In 2009, a health technology assessment of population-based colorectal cancer screening found that biennial FIT at ages 55-74 would be considered the optimal screening strategy in Ireland in terms of potential for reducing incidence and mortality, and cost-effectiveness [10]. The National Cancer Screening Service launched BowelScreen, a national population-based programme, in December 2012. This paper aims to describe the population burden of colorectal cancer by examining trends in colorectal cancer incidence, mortality and survival during 1994-2010, prior to nationwide screening.

## Methods

We examined incidence for 1994-2010 and mortality for 1994-2009 (2009 was the latest year for which mortality data was available at the time of the study). Information on incident cases was abstracted from the National Cancer Registry Ireland (NCRI). The NCRI records all cancers diagnosed in the population usually resident in Ireland through active case finding by tumour registration officers. The completeness of registration for all invasive cancers diagnosed to end 2008 was estimated to be over 97% [11].

The NCR has permission under the Health (Provision of Information) Act 1997 to collect and hold data on all persons diagnosed with cancer in Ireland. The use of that data for research is covered by the Statutory Instrument which established the Registry Board in 1991. All datasets were anonymised prior to analysis.

Site of tumour was recorded according to the International Classification of Diseases 10th revision (ICD10), and analysis included all primary invasive cancers of the colon (C18) and rectum (C19-C20) with a date of diagnosis during 01/01/1994 and 31/12/2010. For each diagnosed cancer, summary stage was derived from primary tumour (T), regional nodes (N) and distant metastasis (M) as recorded in pathology reports or, in the absence of these, from clinical staging, according to TNM 5^th^ edition [12]. Where a patient was classified as MX (“distant metastases cannot be assessed”), the M category was defaulted to “M0” (no distant metastasis). For example, a patient with stage composite T3N1MX was treated as T3N1M0, stage III (Dukes C). Data on treatment received during the first year post-diagnosis was defined as planned first course of tumour directed treatment administered within one year of the diagnosis date (-30 to 365 days) and aimed at removing, destroying or preventing further tumour growth and included four treatment scenarios: (Surgery (Y/N), chemotherapy(Y/N), radiotherapy(Y/N), or not treated [ICD9CM and ICD10-AM]). Analyses of stage and treatment included cases diagnosed during 1995-2009, as this information was incomplete for 2010 cases and unreliable for 1994 cases, the first year of national registration. Colorectal cancer deaths (C18-20) were obtained from the Central Statistics Office (CSO) [13].

Age-standardised rates (ASR) for incidence and mortality were weighted by the European standard population using the direct method [14]. Trends presented as annual percentage change (APC) in ASRs of incidence (1994-2010) and mortality (1994-2009) were calculated using Joinpoint regression [15]. Joinpoint regression was also used to test for linear trends in treatment (1995-2009) and case fraction of early (stage I/II) and late (stage III/IV) disease (1995-2009). For descriptive purposes, age category percentages and treatment category percentages were given for three diagnostic periods: 1995-1999, 2000-2004 and 2005-2009.

In the Irish cancer registry, follow-up of cases is passive, where registered cancer cases are linked to death certificates provided by the CSO [16]. For survival analysis, the dataset was divided into three diagnostic periods: 1994-1998, 1999-2003 and 2004-2008. Survival time was censored at 31 December 2009 to ensure all cases had at least one year follow-up, and because this was the latest date for which death ascertainment was complete. Our manuscript was drafted in late 2013, a point in time when we were confident that all deaths certificates from the CSO were matched to the cancer registry database. Cases which were preceded by another cancer (other than non-melanoma skin cancer) were excluded from survival analysis as were autopsy-only cases, death certificate only cases (DCO), colorectal cancers concurrent with other invasive malignancy and colorectal cancers diagnosed 2009-2010. Relative Survival (RS), the ratio of observed survival among a group of cases to the expected survival among the general population of the same age, sex and country, was computed based on deaths from all causes and using national life-tables [17].

## Results

### Incidence

**Figure 1:**
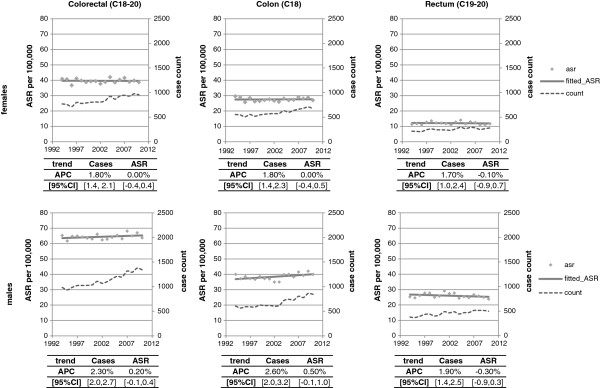
Age standardised incidence rate and incident cases of colorectal cancer by site of primary tumour and sex, 1994-2010.

The colorectal cancer ASR was 63.7 per 100,000 in males and 38.7 per 100,000 in females in 2010. There was little change in the ASR over time (Figure 1) in either sex, or when colon and rectal cancers were considered separately. However, the number of colorectal cancer cases in Ireland increased from 1752 in 1994 to 2298 in 2010, an annual rise of 2.1% (95% CI 1.8, 2.4; p < 0.001). The increase was somewhat higher in males (983 in 1994; 1343 in 2010; APC = 2.3%, 95% CI 2.0, 2.7) than females (769 in 1994; 955 in 2010; APC = 1.8%, 95% CI 1.4, 2.1).

In males, 62% of cases were in the colon; this was 71% in females. Increases in cases were observed in both colon (APC males = 2.6%, 95% CI 2.0, 3.2; APC females = 1.8%, 95% CI 1.4, 2.3) and rectal cancer (APC males = 1.9%, 95% CI 1.0, 2.4; APC females = 1.7%, 95% CI 1.4, 2.5).

### Age distribution

Sixty nine percent of cases in males and 67% in females occurred in those aged ≥65; similar proportions in each sex were diagnosed aged 55-64 (males: 20%; females: 19%) and <55 (males: 11%; females: 14%). Over the three periods 1995-1999, 2000-2004 and 2005-2009 there was no change in the age distribution of either colon or rectal cancer in females or rectal cancer in males (data not shown).

### Stage

**Figure 2:**
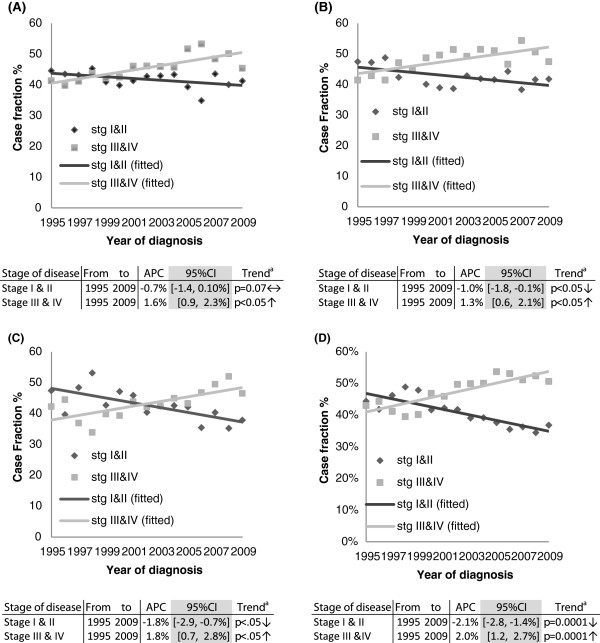
**Case fraction for stage of disease at presentation, by gender and site of tumour for diagnostic period 1994-2009. (A)** COLON- FEMALES; **(B)** COLON MALES; **(C)** RECTUM- FEMALES; **(D)** RECTUM - MALES. ^a^The p-value results are derived from a test of trend. The null hypothesis is that the APC = 0%: Alternative hypothesis is APC ≠ 0%. The APC is the slope of a log-linear regression curve from 1994-2009.

During 1995-2009 early stage (I/II) colon cancers decreased by -1% annually in males (95% CI -1.8%, -0.1%) and in females by -0.7% (95% CI -1.4%, -0.1%). Conversely late stage (III/IV) colon cancers increased by 1.3% in males (95% CI 0.6%, 2.1%) and by 1.6% in females (95% CI 0.9%, 2.3%). Similarly early stage rectal cancers decreased by -2.1% (95% CI -2.8%, -1.4%) in males and -1.8% (95% CI -2.9%, -0.7%) in females, while late stage disease increased significantly (males: APC = 2.0%, 95% CI 1.2%, 2.7%; females: APC = 1.8%, 95% CI 0.7%, 2.8%; Figure 2). Unstaged colon cancers decreased significantly in males by -2.2% (95% CI -4.1%, -0.2%; p-trend <0.05) and by -3.3% in females (95% CI -5.7%, -1.0%; p-trend < 0.05) annually. There was no significant change in unstaged rectal cancers in males (APC 0.6%, 95% CI -2.3%, 1.2%; p-trend = 0.5) or females (APC = 0.2%, 95% CI-2.3%, 2.8; p-trend = 0.8).

### Treatment

**Figure 3:**
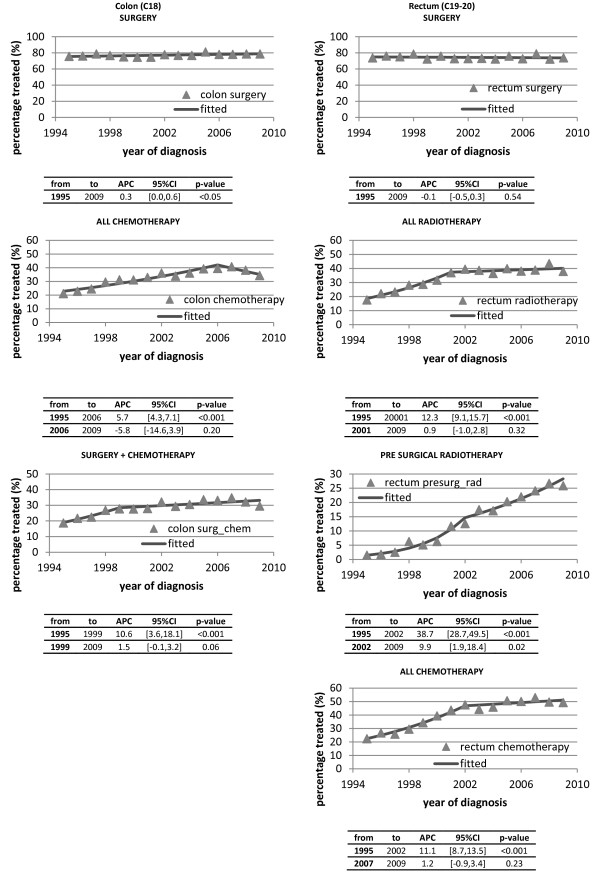
Percentage of patients treated with various modalities 1995-2009.

Use of cancer-directed surgery (i.e. resection) for colon cancer increased from 76% in 1995-1999 to 79% in 2005-2009 (APC 0.3%, 95% CI 0.0, 0.6; p = 0.027), while for rectal cancer there was little change, remaining at 74% over the same period (APC -0.1%, 95% CI -0.5, 0.3; p = 0.54) (Figure 3). Use of chemotherapy for colon cancer rose significant from 21% in 1995 to 40% in 2006, thereafter levelling off to 38% up to 2009(APC = 5.7%, 95% CI 4.3, 7.1; p < 0.001). Similarly, in rectal cancer, chemotherapy use increased significantly from 22% in 1995 to 48% in 2002 (APC = 11.1%, 95% CI 8.7, 13.5; p < 0.001), reaching 49% by 2009 (Figure 3). Use of radiotherapy for rectal cancer increased significantly from 18% in 1995 to 37% in 2001, thereafter levelling off to just under 40% (APC 12.3%, 95% CI 9.1, 15.7; p < 0.001). The proportion of rectal cancer patients who received pre-surgery radiotherapy increased from 2% in 1995 to 13% in 2002 (APC 38.7%, 95% CI 28.7, 49.5; p < 0.001). Thereafter, the proportion receiving this combination increased at a slower rate from 18% in 2003 to 26% in 2009 (APC 9.9%, 95% CI 1.9, 18.4; p = 0.02) (Figure 3).

### Survival

**Figure 4:**
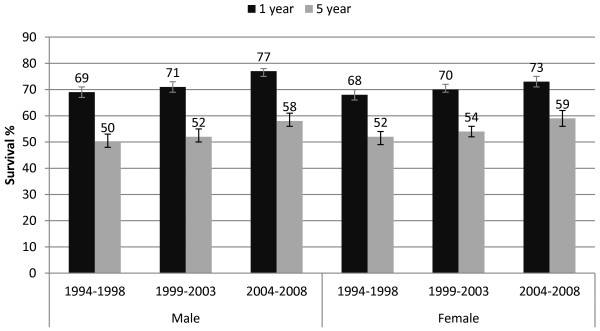
One and five year relative survival for colon cancer for diagnostic periods by sex with 95% confidence intervals.

**Figure 5:**
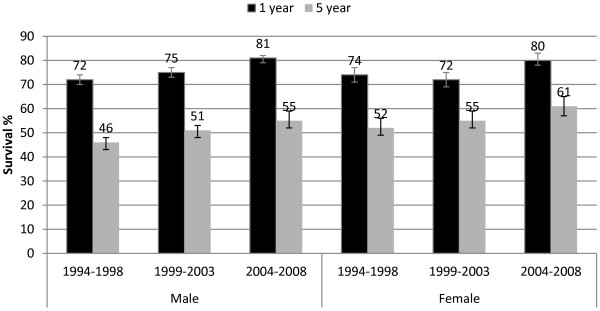
One and five year relative survival for rectal cancer by diagnostic period by sex, with 95% confidence intervals.

Relative survival improved over time for both sexes for colon and rectal tumours. From 1994-1998 to 2004-2008 1-year colon cancer survival in males increased by 8 percentage points to 77% (95% CI 75%, 78%), and in females by 5 percentage points to 73% (95% CI 71%, 75%). Five-year colon cancer survival increased by 8 percentage points to 58% (95% CI 56%, 61%) in males and by 7 percentage points to 59% (95% CI 56%, 62%) in females over the same time (Figure 4). One-year rectal cancer survival improved in males by 9 percentage points to 81% (95% CI 79%, 82%) and in females by 6 percentage points to 80% (95% CI 78%, 83%); 5-year rectal cancer survival in males improved by 9 percentage points to 55% (95% CI 52%, 59%) and in females by 9 percentage points to 61% (95% CI 57%, 65%; Figure 5).

### Mortality

**Figure 6:**
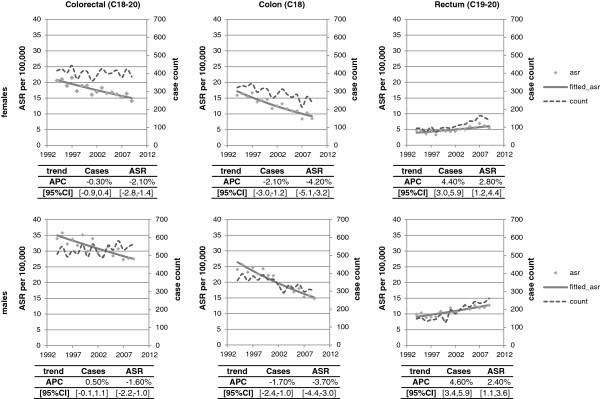
Age standardised mortality rate and number of deaths for colorectal cancer by site of primary tumour and sex, 1994-2009.

In 2005-2009, on average 400 females (255 colon; 145 rectum) and 552 males (313 colon; 239 rectum) died from colorectal cancer annually. Colon cancer deaths declined over time in both sexes (males: 360 in 1994; 302 in 2009; APC = -1.7%, 95% CI -2.4%, -1.0%; females: 321 in 1994; 240 in 2009; APC = -2.1%, 95% CI -3.0%, -1.2%). Rectal cancer deaths rose significantly in males from 148 in 1994 to 262 in 2009 (APC = 4.6%, 95% CI 3.4%, 5.9%) and in females from 94 in 1994 to 141 in 2002 (APC = 4.4%, 95% CI 3.0%, 5.9%).Colorectal cancer age-standardised mortality rates (ASR) decreased by -1.8% (95% CI -2.2%, -1.4%) annually during 1994-2009. Colon cancer ASRs fell in both sexes (males: APC = -3.7%, 95% CI 4.4%, -3.0%; females: APC = -4.2%, 95% CI -5.1%, -3.2%), but rectal cancer ASR (mortality) rose (males: APC = 2.4%, 95% CI 1.1%, 3.6%; females: APC = 2.8%, 95% CI 1.2%, 4.4%; Figure 6).

## Discussion

Over the past 20 years the number cases of colorectal cancer has increased significantly in Ireland; however once adjusted for changes in the age distribution of the population over time the rate has remained stable. Internationally colorectal cancer rates have stabilised in economically developed countries and Ireland is no exception in this regard [18]. In comparison to other European countries, in 2008 Ireland had a higher incidence rate than the EU average and 23% higher than the rate in the United Kingdom [19]. In the European region incidence has increased in males at a greater rate than female incidence during the period 1988 to 2008 [20]. Survival was just below the EU average but similar to the United Kingdom [21]. The improvements in survival reported in this paper were also seen in other European countries during the 1990s and early 2000s [21]. European 5 year survival of colon cancer increased from 54.2% in the period 1999-2001 to 58.1% in 2005-2007, and from 52.1% to 57.6% for rectal cancer over the same period [22]. Although Irish survival improved, it is still lower than the European average [22]. Our data indicates that survival continued to improve for cases diagnosed during 2005-2009. While we did not have detailed information on the dose and intensity of chemotherapy and radiotherapy regimens, better uptake in and application of treatment options during 1995-2009 correlate with the improvement in survival.

### Stage

One of the striking findings of this study was that almost half of cases had relatively late stage at diagnosis (stage III/IV) and, over the period under investigation, the proportion with stage III/IV disease increased from 42% to 50%. The increase in stage III/IV cancers is likely to be as a result of more comprehensive investigation in the peri-operative period, with improvements in imaging and diagnostic methods, resulting in a significant shift in stage allocation from stage I/II to stage III/IV over the years 1995-2009. Another possibility is that the number of nodes taken at resection increased over the period 1995-2009, thereby leading to a situation where the probability of finding a positive node(s) increased commensurately, which would have tipped the balance in favour of stage III/IV over stage I/II according to UICC-TNM, 5th edition. However, we do not have details on node count to support this hypothesis. This question will be addressed in a more comprehensive study of stage migration in colorectal cancer at this registry.

If effective, screening has the potential to change the stage distribution of colorectal cancer in the population. As regards FIT-based screening, which is being implemented in Ireland, Cole et al reported that colorectal cancers were detected at significantly earlier stages in those invited to participate in a screening programme using FIT [23]. In a health technology assessment for Ireland, it was estimated that, by year 10 of a programme, the percentage of cases diagnosed at stages I/II would increase from 46% to 53% and stages III/IV decrease from 54% to 47% [10]. These estimates were based on screening targeted at those aged 55-74 with a best case scenario uptake of 53% (based on the UK experience of FOBT screening) [24]. Similar uptake has been achieved in pilot FIT screening in Ireland [25]. The BowelScreen programme, which has recently commenced, is initially inviting individuals aged 60-69. While the stated intention is to eventually include 55-74 year olds, this is likely to take a number of years due to the development of colonoscopy capacity. Therefore the estimates of potential reductions in late stage disease are very unlikely to be achieved by year 10 of the programme.

### Mortality

In 2008 Ireland ranked midway of 30 European countries in relation to mortality, similar to the EU average but marginally higher than the United Kingdom [19]. Annual decreases in age standardised mortality rates for colorectal cancer in males and females were observed in this study. However this concealed significant increases in the mortality rate for rectal cancers of 2.4% in males and 2.8% in females. Scrutiny of European data reveals that most countries have experienced static mortality rates over the past 15-20 years. However a few, in addition to Ireland, have described increases. These include Spain, with an APC of 3.5% during 1994-2005, Malta with an APC of 5.2% during 1994-2008 and among selected registries in Germany with an APC of 17.1% during 1998-2007 [26]. In terms of potential explanations for these trends, the first that must be considered is whether it might be an artefact of coding of rectal cancer deaths. We have shown that there was a significant decline in the annual death rate for pooled colorectal sites. Yet, there was a steeper decline in the rate of colon deaths, with a compensatory increase in the rate for ‘rectum’ deaths. This suggests that there may have been a subtle shift in death certificate coding allocation from ‘colon’ to ‘rectum’ over the period we have examined. It has long been recognised that physicians tend to report non-specific cancer sites on death certificates; thus, if physicians change how they record cause of death on the death certificate over time, this may induce an apparent change in mortality rates [27]. In 1981, Percy et al reported that misclassification led to over reporting of colon cancer deaths and underreporting of rectal cancer deaths [27]. More recently, in the US, Yin et al reported inaccurate coding of underlying cause of death, with the vast majority of misclassifications being colon cancers incorrectly classified as rectal cancers [28]. Further investigation is warranted to explore the extent and nature of misclassification on death certificates in European countries in recent years, perhaps comparing countries with rising and static rectal cancer mortality rates.

Another possible explanation of the observed increase in rectal cancer mortality is patterns in treatment utilisation. Pre-operative radiotherapy has been recommended for resectable rectal cancer in recent years [29,30] and in line with this the proportion who received pre-operative radiotherapy has increased markedly since 2000, in Ireland and in other countries [31]. However Carsin et al have reported low use of radiotherapy in Ireland (27%) [31] compared to US and EU populations (46%-62%) [32-34]. Moreover, although data from trials suggests that pre-operative use is more effective, a significant proportion treated with radiotherapy in Ireland receive it post-operatively rather than pre-operatively [31]. These observations raise the possibility that underuse of radiotherapy, particularly preoperative radiotherapy, may be a contributor to rectal cancer mortality trends. Moreover, while the current study found that radiotherapy use was continuing to rise, any impact of this on mortality rates will not be seen for several years.

In terms of surgery, evidence-based guidelines have been published in Ireland aimed at standardising surgical management of rectal cancer [30]. An audit of all rectal cancers diagnosed in 2007 found that, while guidelines were in place, best practice was frequently not adhered to [35]. Surgery for rectal cancer can result in significant morbidity if undertaken without appropriate and accurate pre-operative staging. Accurate localisation of the tumour [36-38], use of MRI (magnetic resonance imaging) [39] and ERUS (Endo-rectal ultrasound) [40-42] as diagnostic tools, and recording of accurate pre-operative histological data [43,44], are all essential for successful treatment. However the national audit revealed that there were often inadequate investigations and/or recording of such data [35]. In addition while multi-disciplinary meetings (MDM) have been shown to improve outcomes for rectal cancer [45,46], treatment options were only discussed at MDMs for around half of patients. Moreover patients treated at low volume centres were less likely to be discussed at MDMs and to have neo-adjuvant therapy [35]. Further evidence suggests that comorbidity, rather than age, in elderly rectal cancer patients increases risk of death after surgery [47]. Therefore age alone should not dictate the use of restorative rectal resection [47]. However, our analyses indicate lower use of surgery in elderly than younger patients (≥75; 81%; <75: 92-99%) as well as larger increases in age standardised mortality in those aged 70 and older [13]. These observations, combined with likely under use of best practice, may provide a possible explanation for the observed trends in mortality.

Biennial FIT-based screening in the 55-74 age group in Ireland could reduce colorectal cancers deaths in the population from as early as the second year of the programme [10]. However, as noted earlier, screening is being introduced in those aged 60-69, suggesting that it is likely to take some considerable time to have any impact on the trends in rectal cancer mortality reported here.

## Conclusion

Age standardised incidence has remained static in Ireland over the period 1994-2010, but 1-year and 5-year survival continues to increase in both sexes. The proportion of cases with late stage disease has increased over time, as have mortality rates for rectal cancer. These trends indicate the need for efficient and timely roll-out of BowelScreen. However the narrow age-range at which BowelScreen will operate in the first instance means that the potential benefits of screening, in terms of more advantageous stage distribution and reductions in colorectal cancer incidence and mortality in the population, are unlikely to be achieved in the short-term.

## Competing interests

The authors declare that they have no completing interests.

## Authors’ contributions

NC contributed to interpretation and drafted the manuscript. JMcD carried out statistical analysis. LS conceived of the study and advised on analysis and interpretation. PK advised on analysis and interpretation. JMcD, LS and PK commented on drafts of the manuscript. All authors approved the final version.

## Authors’ information

NC is a PHD student funded under an Irish Cancer Society Scholarship and working with the National Cancer Registry Ireland. NC is registered within the Department of Epidemiology and Public health, University College Cork. JMcD is a researcher and statistical analyst at the National Cancer Registry Ireland. PMK is a research professor within the Department of Epidemiology and Public Health, University College Cork. LS is research manager in the National Cancer Registry Ireland and adjunct professor in the Department of Epidemiology and Public Health, University College Cork.

## Pre-publication history

The pre-publication history for this paper can be accessed here:

http://www.biomedcentral.com/1471-230X/14/92/prepub

## References

[B1] GLOBOCAN IARC Section of Cancer Information (Cited:(17/12/2012) 2008 Available at: http://globocan.iarc.fr

[B2] PDQ® Cancer Information Summary Colorectal Cancer Screening—Health Professional. Date last modified: 09/30/2011 2011 Bethesda, Maryland: National Cancer Institute

[B3] Ouyang DL Chen JJ Getzenberg RH Schoen RE Noninvasive testing for colorectal cancer: a review Am J Gastroenterol 2005 100 6 1393 1403 10.1111/j.1572-0241.2005.41427.x 15929776

[B4] Burch JA Soares-Weiser K St John DJ Duffy S Kleijnen J Westwood M Diagnostic accuracy of faecal occult blood tests used in screening for colorectal cancer: a systematic review J Med Screen 2007 14 3 132 137 10.1258/096914107782066220 17925085

[B5] Zavoral M Suchanek S Zavada F Dusek L Muzik J Seifert B Fric P Colorectal cancer screening in Europe World J Gastroenterol 2009 15 47 5907 5915 10.3748/wjg.15.5907 20014454 PMC2795177

[B6] Benson VS Patnick J Davies AK Nadel MR Atkin W International Colorectal Cancer Screening Network Colorectal cancer screening: a comparison of 35 initiatives in 17 countries Int J Cancer 2008 122 1357 1367 18033685 10.1002/ijc.23273

[B7] Vart G Banzi R Minozzi S Comparing participation rates between immunochemical and guaiac faecal occult blood tests: a systematic review and meta-analysis Prev Med 2012 55 87 92 10.1016/j.ypmed.2012.05.006 22634386

[B8] von Karsa L Patnick J Segnan N Atkins W Halloran S Lansdorp-Vogelaar I Malila N Minozzi S Moss S Quirke P Steele RK Veith M Aabakken L Altenhofen L Ancelle-Park R Antolljak N Antttila A Armaroli P Arrossi S Austoker J Banzi R Bellisario C Blom J Brenner H Bretthauer M Camargo Cancela M Costamagna G Cuzick J Dai M European Colorectal Cancer Screening guidelines Working Group European guidelines for quality assurance in colorectal cancer screening and diagnosis: an overview and introduction to the full Supplement publication Endoscopy 2013 45 1 51 59 23212726 10.1055/s-0032-1325997 PMC4482205

[B9] Levin B Lieberman DA McFarland B Smith RA Brooks D Andrews KS Dash C Giardiello FM Glick S Levin TR Pickhardt P Rex DK Thorson A Winawer SJ American Cancer Society Colorectal Cancer Advisory Group; US Multi-Society Task Force; American College of Radiology Colon Cancer Committee Screening and surveillance for the early detection of colorectal cancer and adenomatous polyps, 2008: a joint guideline from the American Cancer Society, the US Multi-Society Task Force on Colorectal Cancer, and the American College of Radiology Gastroenterology 2008 134 1570 1595 10.1053/j.gastro.2008.02.002 18384785

[B10] Information H Authority Q Health technology assessment (HTA) of a population-based colorectal cancer screening programme in Ireland 2009 Health Information and Quality Authority: Dublin

[B11] National Cancer Registry, Ireland Data quality and completeness at the Irish National Cancer Registry 2012 Cork: National Cancer Registry Ireland [http://www.ncri.ie]

[B12] Sobin LH Wittenkind C International Union Against Cancer (UICC), TNM classification of malignant tumours 1997 5 NY, USA: Wiley-Liss

[B13] Central Statistics Office, Ireland [http://www.cso.ie/en/releasesandpublications/birthsdeathsandmarriages/]

[B14] International Agency for Research on Cancer Cancer Registration: Principles and Methods 1991 Lyon, France: International Agency for Research on Cancer

[B15] Kim HJ Fay MP Feuer EJ Midthune DN Permutation tests for Joinpoint regression with applications to cancer rates Stat Med 2010 19 335 351 Software available at URL: http://surveillance.cancer.gov/joinpoint/ [Accessed Jan 2011] 10649300 10.1002/(sici)1097-0258(20000215)19:3<335::aid-sim336>3.0.co;2-z

[B16] O’Brien KO Comber H Sharp L Completeness of case ascertainment at the National Cancer Registry Ir J Med Sci 2013 doi:10.1007/s11845-013-0993-z 10.1007/s11845-013-0993-z 23955644

[B17] Dickman PW Sloggett M Hills M Hakulinen T Regression models for relative survival Stat Med 2004 23 1 51 64 10.1002/sim.1597 14695639

[B18] Center M Jemal A Ward E International trends in colorectal cancer incidence rates Cancer Epidemiol Biomarkers Prev 2009 18 1688 1694 10.1158/1055-9965.EPI-09-0090 19505900

[B19] European Cancer Observatory (ECO) European Cancer Observatory (ECO) http://eu-cancer.iarc.fr/

[B20] Arnold M Karim-Kos HE Coebergh JW Byrnes G Antilla A Ferlay J Renehan AG Forman D Soerjomataram I Recent trends in incidence of five common cancers in 26 European countries since 1988: analysis of the European Cancer Observatory Eur J Cancer (Epub ahead of print) http://dx.doi.org/10.1016/j.ejca.2013.09.002 10.1016/j.ejca.2013.09.002 24120180

[B21] Verdecchia A Francisci S Brenner H Gatta G Micheli A Mangone L Kunkler I EUROCARE-4 Working Group. Recent cancer survival in Europe: a 2000-02 period analysis of EUROCARE-4 data Lancet Oncol 2007 8 9 784 796 10.1016/S1470-2045(07)70246-2 17714993

[B22] De Angelis R Sant M Coleman MP Francisci S Baili P Pierannunzio D Trama A Visser O Brenner H Ardanaz E Bielska-Lasota M Engholm G Nennecke A Siesling S Berrino F Capocaccia R EUROCARE-5 Working Group Cancer survival in Europe 1999-2007 by country and age: results of EUROCARE-5-a population-based study Lancet Oncol 2014 15 23 34 10.1016/S1470-2045(13)70546-1 24314615

[B23] Cole SR Tucker GR Osborne JM Byrne SE Bampton PA Fraser RJ Young GP Shift to earlier stage at diagnosis as a consequence of the National Bowel Cancer Screening Program Med J Aust 2013 198 6 327 330 10.5694/mja12.11357 23545032

[B24] Weller D Moss S Butler P Campbell C Coleman D Melia J Robertson R English pilot of bowel cancer screening: an evaluation of the second round. Final report to the Department of Health 2006 Edinburgh: University of Edinburgh

[B25] McNamara D Qasim A Lee N Condon C O’Morain C Round one of the Adelaide and Meath Hospital/Trinity College Colorectal Cancer Screening Programme: programme report and analysis based on established international key performance indices Ir J Med Sci 2011 180 549 552 10.1007/s11845-010-0650-8 21264524

[B26] Steliarova-Foucher E O’Callaghan M Ferlay J Masuyer E Forman D Comber H Bray F European Cancer Observatory: Cancer Incidence, Mortality, Prevalence and Survival in Europe Version 1.0 (September 2012) European Network of Cancer Registries, International Agency for Research on Cancer 2013 Available from http://eco.iarc.fr, Accessed February 2013

[B27] Percy C Stanek E Gloeckler L Accuracy of Cancer Death Certificates and Its Effect on Cancer Mortality Statistics Am J Public Health 1981 71 3 242 250 10.2105/AJPH.71.3.242 7468855 PMC1619811

[B28] Yin D Morris CR Bates JH German RR Effect of Misclassified Underlying Cause of Death on Survival Estimates of Colon and Rectal Cancer J Natl Cancer Inst 2011 103 1130 1133 10.1093/jnci/djr207 21697545

[B29] Sauer R Becker H Hohenberger W Rödel C Wittekind C Fietkau R Martus P Tschmelitsch J Hager E Hess CF Karstens JH Liersch T Schmidberger H Raab R for the German Rectal Cancer Study Group Preoperative versus postoperative chemo-radiotherapy for rectal cancer N Engl J Med 2004 351 17 1731 1740 10.1056/NEJMoa040694 15496622

[B30] The Association of Coloproctology of Great Britain and Ireland Guidelines for the Management of Colorectal Cancer 2007 3 [http://www.acpgbi.org.uk/assets/documents/COLO_guides.pdf, Accessed 13/08/2012]

[B31] Carsin A-E Sharp L Cronin-Fenton DP Céilleachair AO Comber H Inequity in colorectal cancer treatment and outcomes: a population-based study Br J Cancer 2008 99 266 274 10.1038/sj.bjc.6604467 18594530 PMC2480963

[B32] Ayanian J Zaslavsky A Fuchs C Guadagnoli E Creech C Cress R O’Connor L West D Allen M Wolf R Wright W Use of adjuvant chemotherapy and radiation therapy for colorectal cancer in a population-based cohort J Clin Oncol 2003 21 1293 1300 10.1200/JCO.2003.06.178 12663717

[B33] Cronin D Harlan L Potosky A Clegg L Stevens J Mooney M Patterns of care for adjuvant therapy in a random population-based sample of patients diagnosed with colorectal cancer Am J Gastroenterol 2006 101 2308 2318 10.1111/j.1572-0241.2006.00775.x 17032196

[B34] Vulto J Louwman W Lybeert M Poortmans P Rutten H Brenninkmeijer S Coebergh JA Population-based study of radiotherapy in a cohort of patients with rectal cancer diagnosed between 1996 and 2000 Eur J Surg Oncol 2007 33 993 997 10.1016/j.ejso.2007.02.019 17400420

[B35] Boyle E Timmons A Al-Akash M Kennedy AM O’Grady H Hill AD Comber H Keane FB The management of rectal cancer in Ireland 2007 – room for improvement? Surgeon 2011 9 179 186 10.1016/j.surge.2010.09.006 21672656

[B36] Piscatelli N Hyman N Osler T Localising colorectal cancer by colonoscopy Arch Surg 2005 140 932 935 10.1001/archsurg.140.10.932 16230540

[B37] Schoellhammer HF Gregorian AC Sarkisyan GG Petrie BA How important is rigid proctosigmoidoscopy in localizing rectal cancer? Am J Surg 2008 196 6 904 908 10.1016/j.amjsurg.2008.08.005 19095107

[B38] Wibe A Møller B Norstein J Carlsen E Wiig JN Heald RJ Langmark F Myrvold HE Søreide O Norwegian Rectal Cancer Group A national strategic change in treatment policy for rectal cancer implementation of total mesorectal excision as routine treatment in Norway. A national audit Dis Colon Rectum 2002 45 7 857 866 10.1007/s10350-004-6317-7 12130870

[B39] Butch RJ Stark DD Wittenberg J Tepper JE Saini S Simeone JF Mueller PR Ferrucci JT Jr Staging rectal cancer by MR and CT Am J Roentgenol 1986 146 11 55 60 10.2214/ajr.146.6.1155 3486559

[B40] Muthusamy VR Chang KJ Optimal methods for staging rectal cancer Clin Cancer Res 2007 13 Suppl. 2 6877s 6881s 18006793 10.1158/1078-0432.CCR-07-1137

[B41] Akasu T Kondo H Moriya Y Sugihara K Gotoda T Fujita S Muto T Kakizoe T Endorectal ultrasonography and treatment of early stage rectal cancer World J Surg 2000 24 1061 1068 10.1007/s002680010151 11036283

[B42] Garcia-Aguilar J Pollack J Lee SH Hernandez de Anda E Mellgren A Wong WD Finne CO Rothenberger DA Madoff RD Accuracy of endorectal ultrasonography in preoperative staging of rectal tumors Dis Colon Rectum 2000 45 10 15 11786756 10.1007/s10350-004-6106-3

[B43] Dukes C Histological grading of rectal cancer (section of pathology) Proc R Soc Med 1937 30 4 371 376 19990989 10.1177/003591573703000412 PMC2076469

[B44] Bloom HJ Richardson WW Histological grading and prognosis in breast cancer Br J Cancer 1957 11 359 377 10.1038/bjc.1957.43 13499785 PMC2073885

[B45] Obias VJ Reynolds HL Multidisciplinary teams in the management of rectal cancer Clin Colon Rectal Surg 2007 20 3 143 147 10.1055/s-2007-984858 20011195 PMC2789504

[B46] MacDermid E Hooton G MacDonald M McKay G Grose D Mohammed N Porteous C Improving patient survival with the colorectal cancer multi-disciplinary team Colorectal Dis 2009 11 3 291 295 10.1111/j.1463-1318.2008.01580.x 18477019

[B47] Manceau G Karoui M Werner A Mortensen N Hannoun L Comparative outcomes of rectal cancer surgery between elderly and non-elderly patients: a systematic review Lancet Oncol 2012 13 e525 e536 10.1016/S1470-2045(12)70378-9 23182193

